# Variability in documentation of neurological and psychiatric examinations among emergency department patients with behavioral health-related presentations: a retrospective study

**DOI:** 10.3389/fpsyt.2026.1799470

**Published:** 2026-04-10

**Authors:** Heba Mesbah, Payton Kim, Jide Oluwadare, Abdulelah Almanie, Inaara Malick, Nidal Moukaddam

**Affiliations:** 1 Emergency Medicine Department, Baylor College of Medicine, Houston, TX, United States; 2 Baylor College of Medicine, Houston, TX, United States; 3 Psychiatry and Behavioral Sciences Department, Baylor College of Medicine, Houston, TX, United States

**Keywords:** disparities, documentation, electronic medical record (EMR), emergency psychiatry, health equity

## Abstract

**Background:**

Emergency departments (EDs) serve as a critical safety net for individuals experiencing acute behavioral health crises, a population that faces substantial medical morbidity and well-documented disparities in healthcare delivery. Thorough physical and neuropsychiatric assessment is essential in this setting to identify medical conditions that may mimic or exacerbate psychiatric symptoms. Incomplete documentation of these assessments may reflect gaps in care processes and represent a potential marker of inequity.

**Objective:**

To characterize the completeness of documented neurological and psychiatric examinations among adult ED patients presenting with primary behavioral health-related chief complaints and to assess whether documentation patterns suggest persistent gaps in standardized evaluation.

**Methods:**

We conducted a retrospective electronic medical record review of adult patients presenting to a large, urban academic ED between May 2020 and May 2021 with behavioral health-related chief complaints requiring medical clearance prior to psychiatric evaluation. Documentation of neurological and psychiatric examination components was systematically abstracted using predefined operational definitions.

**Results:**

Of 1,613 screened encounters, 507 met inclusion criteria (mean age 39.1 ± 14 years; 66.7% male; 49.7% African American). Suicidal ideation was the most common presenting complaint (49.9%), and 55.0% of patients presented voluntarily. A general neurological or mental status examination was documented in 94.5% of encounters; however, specific neurological components such as Glasgow Coma Scale (9.3%) and deep tendon reflexes (1.4%) were infrequently recorded. Psychiatric examinations were documented in 63.3% of cases, with behavioral observations most commonly reported and cognition and memory least frequently assessed.

**Conclusions:**

Documentation of neurological and psychiatric examinations for ED patients with primary behavioral health presentations remains inconsistent, particularly for specific examination components. When documentation is used as a surrogate for care processes, these findings suggest variability in the thoroughness of evaluation for a vulnerable population. Establishing standardized, evidence-based expectations for neuropsychiatric assessment and documentation in the ED may represent an important step toward improving patient safety and promoting equity in emergency psychiatric care.

## 1 Introduction

Emergency departments (EDs) play a central role in the care of individuals experiencing acute behavioral health crises and function as a critical safety net for populations disproportionately affected by social and structural disadvantage. Patients presenting with psychiatric symptoms frequently experience higher rates of medical comorbidity, premature mortality, and barriers to accessing longitudinal mental health care, resulting in increased reliance on emergency services (1–4). These challenges are compounded by structural stigma, socioeconomic instability, and limited outpatient resources, all of which contribute to disparities in both access to care and clinical outcomes.

For patients presenting with behavioral health-related complaints, a timely and thorough physical and neurological examination is a cornerstone of safe emergency care. Psychiatric symptoms may arise from or coexist with underlying medical conditions, including infection, intoxication, metabolic disturbances, trauma, and neurological disease (4–6). Failure to adequately assess for these conditions may lead to missed or delayed diagnoses, inappropriate disposition, and preventable harm. Individuals with serious mental illness are particularly vulnerable, as they have a higher prevalence of undiagnosed or undertreated medical illness compared to the general population (1–3).

Despite this risk, behavioral health patients are susceptible to diagnostic overshadowing, in which physical symptoms or abnormal findings are attributed to a psychiatric condition, and diagnostic anchoring, whereby early assumptions prematurely truncate medical evaluation. These cognitive and structural factors may influence not only clinical decision-making but also the thoroughness of physical examination and its documentation in the electronic medical record (EMR). Documentation completeness is increasingly recognized as a meaningful process metric, reflecting both the delivery and visibility of care, and may offer insight into inequities affecting vulnerable populations.

A pilot study conducted at our institution in 2016 identified concerning deficiencies in emergency physician documentation of neurological and psychiatric examinations among patients presenting with behavioral health-related complaints, with physical reassessments often absent despite prolonged ED length of stay (7). However, the limited sample size constrained interpretation and precluded broader conclusions regarding whether these findings reflected isolated practice variation or a more persistent, systemic pattern.

To address this gap, we conducted a large-scale retrospective study to examine documentation practices for neurological and psychiatric examinations among adult ED patients presenting with primary behavioral health-related chief complaints. By focusing on encounters in which psychiatric symptoms were the sole presenting concern and controlling for factors that may reasonably limit examination, this study aims to characterize the extent and persistence of documentation variability. We hypothesize that incomplete documentation of neuropsychiatric assessments represents a measurable care process gap that may have implications for patient safety and equity in emergency psychiatric care. Establishing standardized expectations for assessment and documentation may be a critical step toward ensuring consistent, high-quality evaluation for this high-risk population.

## 2 Methods

### 2.1 Study design and setting

We conducted a single-center, retrospective observational cohort study using electronic medical record (EMR) data from an urban, academic Level I trauma center in the United States. The emergency department has an annual census of approximately 88,000 visits and includes a dedicated psychiatric emergency service. The study period spanned from May 1, 2020, to May 30, 2021.

This study was approved by the Institutional Review Board, with a waiver of informed consent due to its retrospective design. Reporting follows the Strengthening the Reporting of Observational Studies in Epidemiology (STROBE) guidelines.

### 2.2 Case identification and participant selection

Patients were identified through the Epic EMR system (Epic Systems Corporation, Verona, WI). We generated a list of all adult ED encounters during the study period with primary behavioral health-related chief complaints, identified using the following ICD-10 codes: suicidal ideation (R45.85), homicidal ideation (R45.850), agitation (R45.1), aggression (R45.6), and bizarre behavior (R46.2).

Encounters were screened consecutively.

#### 2.2.1 Inclusion criteria

- Age ≥ 18 years
- Presentation to the ED with a primary behavioral health-related chief complaint
- Requirement for medical evaluation or clearance prior to psychiatric assessment

#### 2.2.2 Exclusion criteria

- Pregnancy
- Incarceration at time of presentation
- Evidence of intoxication on arrival, as determined by clinicians upon physical exam or by patient self-reporting upon history taking by clinician
- Presence of a concurrent physical presenting complaint (e.g. chest pain; trauma; focal neurologic symptoms, including weakness, numbness, or vision changes), as reported by patient at triage and was confirmed by reviewing the medical notes and final ED diagnosis
- Incomplete or unavailable EMR data
- Patients presenting with both psychiatric symptoms and active medical complaints were excluded to isolate encounters in which psychiatric symptoms were the sole presenting concern, thereby minimizing clinical justification for omitting neuropsychiatric examination documentation.

### 2.3 Data abstraction and variables

Data abstraction was performed using a standardized data collection instrument developed *a priori*. Extracted variables included patient demographics, chief complaint, voluntary versus involuntary presentation status, ED disposition, laboratory testing, and documentation of neurological and psychiatric examinations.

#### 2.3.1 Documentation definitions

Documentation was considered present if recorded in either structured EMR templates or free-text provider notes.

Neurological examination documentation was defined using a two-tiered approach:

General neurological or mental status statement, as documented via an structured EPIC template used in this ED with checkboxes including “neurologically intact” or “alert and oriented,” and

Specific neurological examination components outlined in the template, including:

Alertness and orientation: checkbox for “oriented x3” and a free-text box
Glasgow Coma Scale (GCS): options to select a value 1–4 for “Eye”, a value 1–5 for “Verbal”, and a value 1–6 for “Motor” or a checkbox for “GCS 15” and a free-text box
Cranial nerve assessment: checkbox for “cranial nerves intact” and free-text option
Motor examination: options to mark “weakness”, “tremor”, “atrophy”, “abnormal tone”, “seizure activity”, and “pronator drift” as positive or negative; and a free-text box
Coordination and gait: checkbox for “coordination intact”; options to mark “Romberg”, “abnormal coordination”, “abnormal finger-to-nose”, “abnormal heel-to-shin”, and “impaired RAM” as positive or negative; and a free text box
Deep tendon reflexes: checkbox for “DTRs normal and symmetric”; option to mark “abnormal DTRs” as positive or negative; options to select a value 0–4 for strength of right and left triceps, biceps, brachioradialis, patellar, and achilles reflexes; and a free-text box

Psychiatric examination documentation included any recorded assessment of:

Mood and affect: checkboxes for “normal mood” and “normal affect”; options to mark mood as “anxious”, “elated”, “blunt”, “angry”, “inappropriate”, “depressed”, “labile”, “flat”, and “tearful”; and a free-text box
Thought content: checkbox for “normal thought content”; options to mark “paranoid”, “homicidal”, “delusional”, “suicidal”, “plan of suicide”, and “plan of homicide” as positive or negative; and a free-text box
Behavior: checkboxes for “normal behavior”, “cooperative”, and “uncooperative”; options to mark “agitated”, “slowed”, “aggressive”, “withdrawn”, “hyperactive”, and “combative” as positive or negative; and a free-text box
Insight and judgment: checkbox for “normal judgement”; options to mark “impulsive” and “inappropriate” as positive or negative; and a free-text box
Cognition and memory: checkboxes for “normal cognition” and “normal memory”; options to mark “impaired cognition”, “impaired memory”, “impaired recent memory”, and “impaired remote memory” as positive or negative; and a free-text box
Formal or semi-formal mental status examinations: Mini-Mental State Examination documented or equivalent free-text alternatives

### 2.4 Chart review procedures and reliability

Two trained reviewers independently abstracted data from each encounter. Reviewers were blinded to study hypotheses. Discrepancies were resolved by consensus with a third investigator.

To assess abstraction reliability, a 10% random sample of charts was independently reviewed by both abstractors. No discrepancies were identified in this subset and inter-rater percent agreement was found to be 100%.

### 2.5 Outcomes

The primary outcome was the proportion of encounters with documented neurological and psychiatric examination components.

Secondary outcomes included the frequency of documentation for individual examination elements and comparison with previously reported institutional documentation patterns.

### 2.6 Statistical analysis

Descriptive statistics were used to summarize patient demographics, clinical characteristics, and documentation frequencies. Continuous variables are reported as means with standard deviations, and categorical variables as counts and percentages.

Comparisons with prior institutional data were exploratory. Statistical analyses were conducted using STATA version 18.0 (StataCorp, College Station, TX). Statistical significance was defined as a two-sided *p* value < 0.05.

## 3 Results

**Figure 1:**
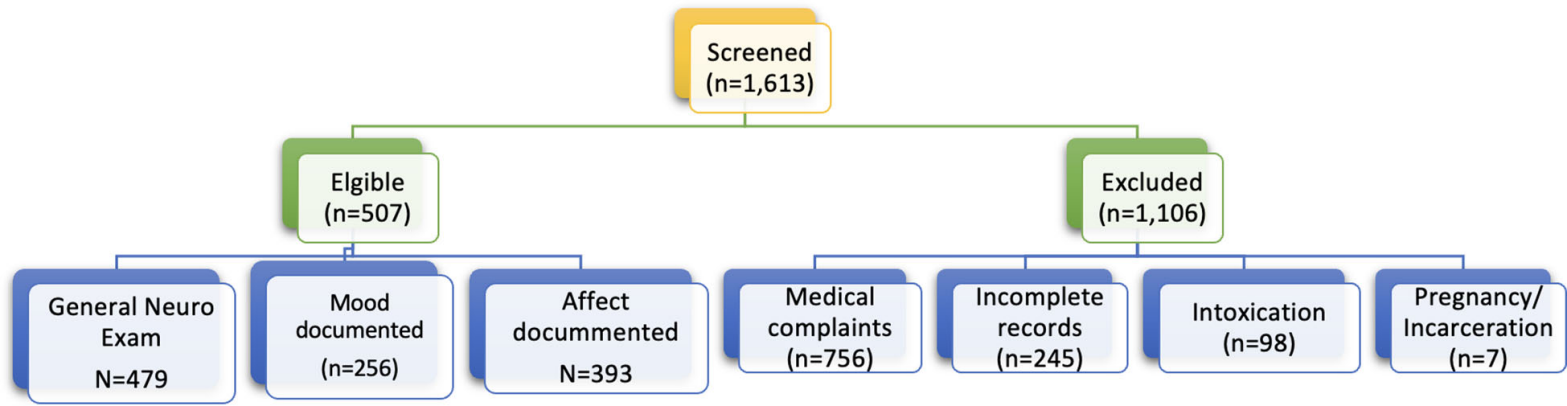
STROBE diagram reporting flow of participants through the study.

A total of 1613 patients were screened, of which 507 (31.4%) met the inclusion criteria (**Figure 1**). Their mean (SD) age was 39.1 (0.6) years, and 66.67% were male. The cohort comprised 49.7% African American patients, 28.6% white patients, and 18.2% Hispanic patients. Suicidality was the most common complaint (49.9%). A total of 55.0% presented to the ED voluntarily, and 89.9% underwent laboratory tests. The ED dispositions included discharge (61.9%), psychiatric unit admission (35.5%), and medical unit admission (2.6%) (**Table 1**).

**Table 1:** shows the characteristics of the patients in our cohort.

Parameter	N (%)
Gender	
· Male	(66.7)
· Female	(33.1)
· Transgender	(0.2)
Race	
· African American	252 (49.7)
· Asian	5 (1.0)
· Hispanic	92 (18.1)
· White	145 (28.6)
· Others	13 (2.6)
Chief complaints	
· Suicidal ideation	253 (49.9)
· Homicidal ideation	130 (25.6)
· Auditory or visual hallucinations	63 (12.43)
· Aggression	36 (7.1)
· Others (paranoia, mania, anxiety, depression)	25 (4.9)
Status	
· Voluntary	279 (55.0)
· Involuntary	228 (45.0)
ED disposition	
· Discharged	314 (61.9)
· Admitted to a psychiatric unit	180 (35.5)
· Admitted to the medicine unit	13 (2.6)

**Figure 2:**
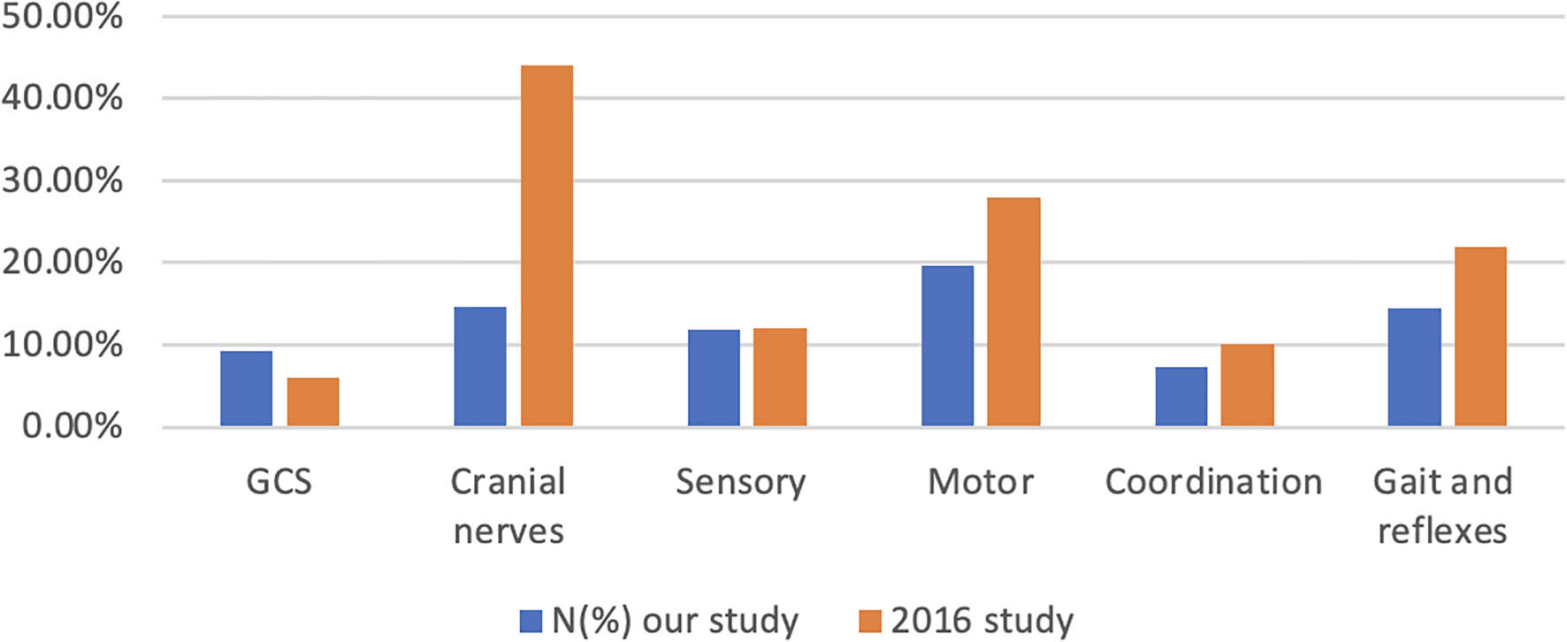
Documentation rates (%) for each of the neurological exam components in both 2016 and the current study.

General neurological examination and mental status were noted in 94.48% of cases. However, the Glasgow Coma Scale (GCS) score was reported in only 9.27% of the patients. Reflexes were the least frequently documented elements of examination by emergency physicians (1.38%) (**Figure 2**).

**Figure 3:**
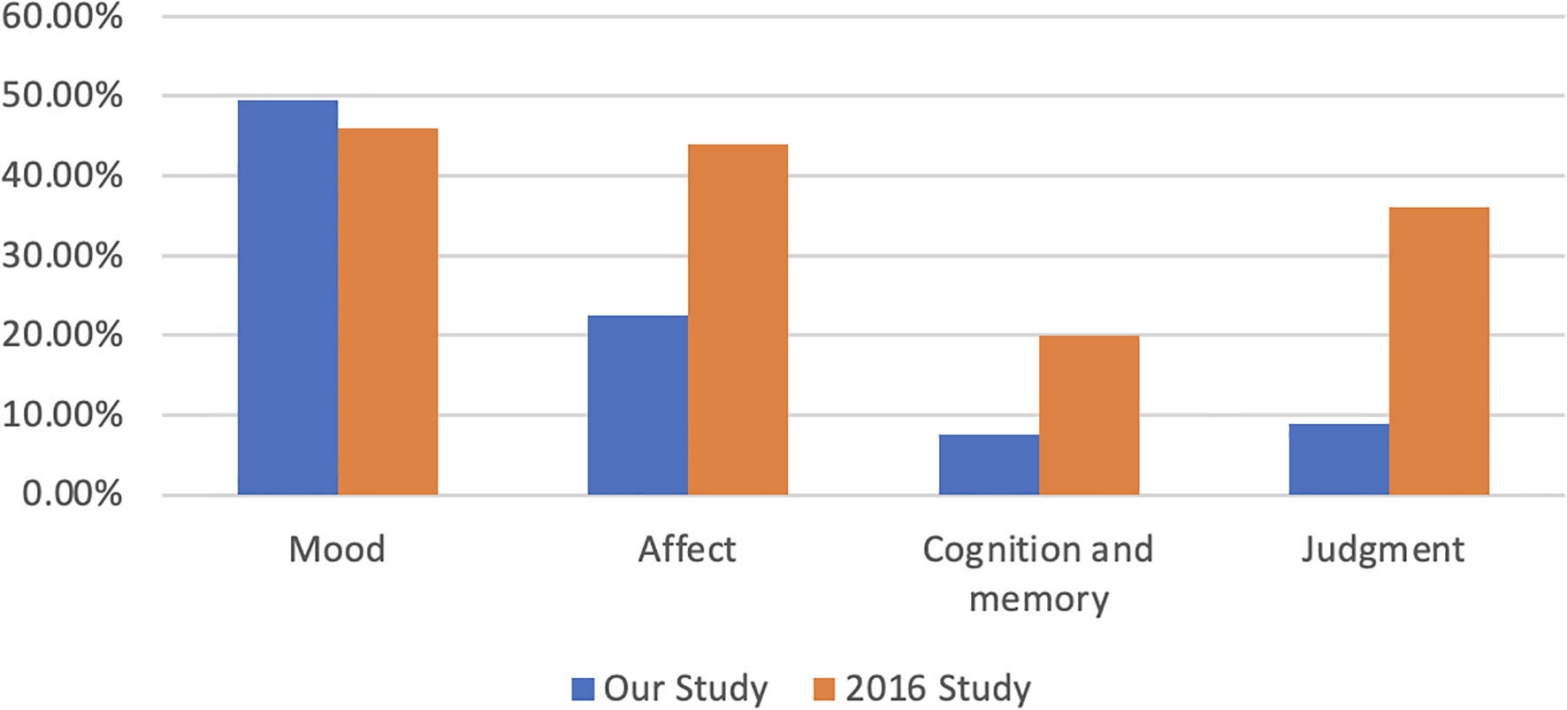
Documentation rates (%) for each of the psychiatric exam components in both 2016 and the current study.

A total of 321 (63.31%) psychiatric examinations were documented, followed by thought content, 291 (57.4%). The least documented elements were cognition and memory, 7.5%. **Figure 3** showed the differences between some of the psychiatric examinations between the two studies.

However, no statistically significant difference was observed in the documentation percentages of neurological physical examinations or psychiatric examinations between 2016 and 2022, with P-values of .761 and .308, respectively. Nevertheless, data for the general neurological examination and the presence or absence of neurological deficits from 2016 onwards were unavailable.

## 4 Discussion

In this retrospective study of adult emergency department (ED) patients presenting with primary behavioral health-related chief complaints, we identified persistent variability in the documentation of neurological and psychiatric examinations. While a general neurological or mental status assessment was documented in most encounters, specific components of neurological and psychiatric examinations were recorded far less consistently. Per recommendations from the American Association for Emergency psychiatry task force, “thorough history and physical examination, including vital signs and mental status examination, are the minimum necessary elements in the evaluation of psychiatric patients” (8). However, these findings suggest that, even within a population for whom neuropsychiatric assessment should be central to care, documentation of key examination elements may often remain incomplete.

This work builds upon a prior institutional review conducted in 2016 that first raised concerns regarding documentation practices for emergency psychiatric patients. Although near-universal documentation of a general mental status or neurological statement was observed in the current cohort, the low frequency of documented examination components—such as Glasgow Coma Scale scoring, reflexes, gait, and cognitive assessment—indicates that comprehensive neuropsychiatric evaluation may not be consistently captured in the medical record. Importantly, comparisons with earlier institutional data did not demonstrate statistically significant improvement in documentation rates over time, suggesting that this pattern may be persistent rather than transient.

Incomplete documentation is not inherently synonymous with incomplete care; however, documentation serves as the primary visible record of clinical assessment and decision-making. When used as a surrogate measure of care processes, variability in documentation may raise concern for inconsistent evaluation standards applied to patients with behavioral health presentations. This is particularly relevant in the ED, where psychiatric symptoms can reflect underlying medical or neurological pathology, including infection, metabolic derangement, intoxication, or central nervous system disease (7). New-onset psychosis, agitation, or suicidal ideation cannot be reliably distinguished from organic illness on history alone, underscoring the importance of systematic neuropsychiatric examination even in the absence of overt physical complaints.

Our findings are consistent with prior literature demonstrating lower rates of documented neurological examination among patients presenting with psychiatric complaints compared with those presenting with primarily neurological conditions (9). This pattern may align with the phenomenon of diagnostic overshadowing, in which physical findings or medical risk are implicitly deprioritized once a psychiatric explanation is presumed (10). While diagnostic overshadowing has traditionally been discussed in relation to diagnostic error, our data suggest it may also manifest at the level of examination and documentation practices. Such processes may be further influenced by time pressure, ED crowding, behavioral complexity, and clinician discomfort, all of which disproportionately affect patients presenting with mental health crises (11).

In terms of equity, this variability is noteworthy. Individuals with serious mental illness experience higher rates of medical comorbidity, premature mortality, and barriers to accessing preventive and longitudinal care (11, 12). The ED may function as their primary point of contact with the healthcare system. In this context, it is possible that inconsistent documentation of physical and neuropsychiatric assessments may represent a structural vulnerability that compounds existing disparities. Rather than reflecting individual clinician intent, these patterns may arise from system-level norms, workflow constraints, and implicit prioritization of chief complaints within emergency care delivery.

Recent changes in ED documentation practices may further contribute to this issue. Shifts toward documentation models that emphasize medical decision-making over comprehensive physical examination for billing purposes may unintentionally obscure the extent of clinical assessment performed, particularly for patients whose presentations are perceived as primarily psychiatric. While these changes aim to reduce documentation burden, they may also limit the ability to assess consistency and equity in care delivery through chart review, reinforcing the importance of defining evidence-based expectations for examination and documentation in this population.

It is also important to note that marginalized populations, particularly racial and ethnic minorities and those with lower socioeconomic status, disproportionately use emergency departments for psychiatric care (13), and as a result gaps in psychiatric care in the ED may disproportionately affect these populations. Further exploration of the demographics of psychiatric documentation disparities represents an important area for potential further study and research.

Importantly, the goal of this work is not to prescribe a uniform or exhaustive neurological examination for all behavioral health presentations. Rather, our findings highlight what may be the absence of a shared, standardized framework defining what constitutes an adequate neuropsychiatric assessment in the ED for patients presenting with psychiatric symptoms alone. Establishing such standards—supported by evidence, specialty consensus, and feasibility—may improve both patient safety and transparency of care processes. Structured documentation tools, clinical decision support, and targeted training represent practical strategies to improve consistency while respecting clinical judgment and workflow constraints (10, 14, 15).

### 4.1 Limitations

This study has several limitations. First, its retrospective design relies on EMR documentation, which may not fully reflect the examinations performed at the bedside. As such, our findings describe documentation practices rather than direct measures of clinical care, which may be inaccurate in cases where an exam components may have been documented without being performed. Second, the study was conducted at a single academic center with a dedicated psychiatric emergency service, which may limit generalizability to other settings.

The study period coincided with the COVID-19 pandemic, a time marked by increased ED strain, altered patient presentation patterns, and staffing challenges. Although clinical clearance processes did not formally change during this period, pandemic-related pressures may have influenced documentation behavior in ways that are difficult to quantify, but may represent an opportunity for further study in the post-pandemic period.

Additionally, by excluding patients with concurrent medical complaints or intoxication, we intentionally focused on encounters in which psychiatric symptoms were the sole presenting concern. While this approach strengthens internal validity for examining documentation expectations, it may reduce applicability to the broader population of ED psychiatric patients. Finally, there is no universal consensus regarding the necessary scope of neurological examination for specific psychiatric presentations, limiting the ability to define an absolute standard against which documentation completeness can be judged.

## Data Availability

The raw data supporting the conclusions of this article will be made available by the authors, without undue reservation.
